# Design and Fabrication of a Flexible Gravimetric Sensor Based on a Thin-Film Bulk Acoustic Wave Resonator

**DOI:** 10.3390/s23031655

**Published:** 2023-02-02

**Authors:** Giovanni Niro, Ilaria Marasco, Francesco Rizzi, Antonella D’Orazio, Marco Grande, Massimo De Vittorio

**Affiliations:** 1 Department of Electrical and Information Engineering, Politecnico di Bari, 70125 Bari, Italy; 2 Center for Biomolecular Nanotechnologies, Istituto Italiano di Tecnologia, 73010 Arnesano, Italy; 3 Department of Engineering and Innovation, Università del Salento, 73100 Lecce, Italy

**Keywords:** wearable device, FBAR, thin-film acoustic wave resonator, MEMS, aluminium nitride, AlN, gravimetric sensor

## Abstract

Sensing systems are becoming less and less invasive. In this context, flexible materials offer new opportunities that are impossible to achieve with bulky and rigid chips. Standard silicon sensors cannot be adapted to curved shapes and are susceptible to big deformations, thus discouraging their use in wearable applications. Another step forward toward minimising the impacts of the sensors can be to avoid the use of cables and connectors by exploiting wireless transmissions at ultra-high frequencies (UHFs). Thin-film bulk acoustic wave resonators (FBARs) represent the most promising choice among all of the piezoelectric microelectromechanical system (MEMS) resonators for the climbing of radio frequencies. Accordingly, the fabrication of FBARs on flexible and wearable substrates represents a strategic step toward obtaining a new generation of highly sensitive wireless sensors. In this work, we propose the design and fabrication of a flexible gravimetric sensor based on an FBAR on a polymeric substrate. The resonator presents one of the highest electromechanical coupling factors in the category of flexible AlN-based FBARs, equal to 6%. Moreover, thanks to the polymeric support layer, the presence of membranes can be avoided, which leads to a faster and cheaper fabrication process and higher robustness of the structure. The mass sensitivity of the device was evaluated, obtaining a promising value of 23.31 ppm/pg. We strongly believe that these results can pave the way to a new class of wearable MEMS sensors that exploit ultra-high-frequency (UHF) transmissions.

## 1. Introduction

The industry of electronics has been drastically revolutionised over the years. The need for smaller devices has resulted in highly miniaturised chips, opening the door to new applications that were unimaginable with the older technologies [1]. In the Internet of Things paradigm, objects have become smart and connected by telecommunication networks [2]. Digital technologies that are used for health monitoring were applied on the skin or even inside of our bodies, resulting in wearable technologies [3,4].

In this context, flexible and stretchable electronics can address scenarios in which the standard bulky and rigid materials cannot be applied in the attachment of systems to the human body, which is characterised by non-flat surfaces [5]. Moreover, unlike flexible materials, rigid chips are brittle and cannot withstand stresses and deformations.

Several works in the literature have reported flexible and wearable sensors [6,7,8,9]; nevertheless, the integration with electronics at radio frequencies is still an issue to overcome. Indeed, the integration of sensors with ultra-high frequencies (UHFs) is a strategic step forward in wearable applications for facilitating integration with wireless systems, overcoming the use of cables and connectors, and enhancing the sensitivity of the devices [10,11].

For radio-frequency (RF) integration, among the types of microelectromechanical (MEM) resonators, thin-film bulk acoustic wave resonators (FBARs) offer the highest working frequencies combined with the lowest footprints. FBARs confine thickness-extension waves in a vertical cavity formed by a piezoelectric material sandwiched between two electrodes. A reflecting layer at the bottom edge is used to limit the standing wave to the piezoelectric region [12]. The piezoelectric material represents the core of such resonators, where the phase velocity of the mechanical wave and the thickness of the material are the key factors for determining the working frequency of a device. By thinning the cavity region, the resonance can be scaled up to the gigahertz (GHz) range.

In this regard, there are several materials suitable for this scope. Lithium niobate (LiNbO_3_) has been used for decades with optimal results [13,14]; however, it is characterised by low phase velocities that comport thinner piezoelectric layers and the fragility of the structures. In contrast, zinc oxide (ZnO) is a promising choice for thin-film resonators because its phase velocities cause the working frequency to be in the GHz range with layers that are a few micrometres thick at the cost of higher dielectric losses [15,16].

Among other piezoelectric materials, aluminium nitride (AlN) presents the highest phase velocities in the literature and very low losses. By exploiting sputtering to obtain high-quality thin-film coatings on flexible substrates, AlN represents the best choice for applying piezoelectricity in RF integration for wearable systems [17,18].

In the literature, several works have demonstrated the feasibility of AlN-based sensing platforms. Indeed, thanks to their very high mass sensitivities, these resonators can be exploited for the development of a huge variety of sensors for antigen detection [19,20], sweat analysis [17], or humidity sensing [21].

In this scenario, the fabrication of flexible FBARs with a demonstrated mass sensitivity allows the development of highly sensitive sensing platforms for wearable and ingestible applications.

The authors of [22] proposed the fabrication of FBARs on thin and flexible silicon substrates. However, the presence of air membranes under the active regions made the structures brittle [23].

Several works reported the fabrication of FBARs on flexible polyimide membranes by using a silicon donor wafer and transfer printing on a flexible substrate [21,24,25]. The drawback of this approach was the necessity of two parallel processes: one for the structuring of the FBAR and another for the fabrication of the cavities on the flexible substrate; this doubled the complexity and costs.

The authors of [26] detailed a planar topology for resonators. However, the resonance was strongly influenced by the shape of the electrodes, thus increasing the complexity of design and fabrication.

Another solution that sped up the fabrication process was reported in [27], where a resonator was fabricated directly on a thick polyimide layer that acted as a reflective surface, thus eliminating the presence of membranes. The resonators presented a good trade-off between performance and cost, but they were fabricated by using ZnO, which is not suitable for ultra-high frequencies (UHFs).

The same approach was applied to an AlN-based FBAR in [28]. Nevertheless, the working frequency of the resonator was 1.5 GHz with a quite low Q-factor of 157 due to the penetration of the solvent under the substrate during the fabrication processes, and the mass sensitivity of the device was not proven.

In this work, we propose a gravimetric mass sensor made with an AlN-based flexible FBAR on an 8 μm thick polyimide substrate whose resonance is at 2.55 GHz. The resonator was designed by using a finite element method (FEM) solver and fabricated using three lithographic processes. Unlike in [28], the working frequency was scaled up to increase the sensitivity, and the sacrificial layer was protected with the anchor of the polyimide at the edges of the silicon wafer through an adhesion promoter. Due to this step, solvents could not penetrate under the FBAR stack during the fabrication. Therefore, the quality of the materials and the repeatability of the process were both increased. The device was characterised and showed an optimal agreement with simulations, and it presented a Q-factor of 218 (40% higher than in [28]) together with an optimal electromechanical coupling factor of 6% (20% higher than in [25]).

Finally, the mass sensitivity of the sensor was proven by spin-coating different amounts of polymethylmethacrylate (PMMA) on the active region of the resonator. The sensor demonstrated a linear decrease in the anti-resonance frequency with the increase in the mass loading, with a very high responsivity of 23.31 ppm/pg.

## 2. Design of the Resonator

**Figure 1:**
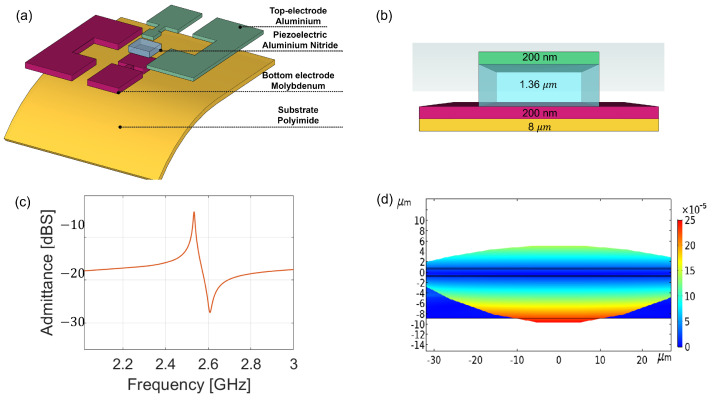
(**a**) Breakdown of the proposed device; (**b**) cross-section of the resonator; (**c**) simulated admittance; (**d**) modal shape of the mechanical travelling wave.

A breakdown of the device’s components is detailed in Figure 1a. The resonator was formed by a piezoelectric AlN layer placed between the top aluminium and the bottom molybdenum electrodes. The stack was placed on an 8 μm thick polyimide substrate. The thicknesses of all the sections are reported in Figure 1b. The molybdenum and aluminium layers had a thickness of 200 nm, while the piezoelectric height was equal to 1.2 μm. The most important layer was the piezoelectric layer, which drove the resonance as detailed in the equation below:

(1) $$f_{r}=v_{p}/\lambda =\frac{v_{p}}{2d}$$

where $f_{r}$ is the resonant frequency, λ is the wavelength of the mechanical standing wave, $v_{p}$ is the acoustic phase velocity, and *d* is the thickness of the piezoelectric layer. The stack was designed by using a solver based on the finite element method (FEM), which was developed by using the electrostatic and mechanical domains offered by Comsol Multiphysics. In particular, the cross-section was defined as detailed in Figure 1b. The input signal was applied to the top electrode, while the ground was at the bottom. The domain was truncated by using a perfectly matched layer and fixed-constraint boundary conditions. The geometry was discretised by using a structured mapped mesh with a minimum size equal to λ/20, where λ is the resonating mechanical wavelength (2.4 μm). Finally, the mechanical and electrical responses of the resonator were evaluated in the frequency-domain solver by using the direct MUMPS solver.

The admittance is reported in Figure 1c. It was possible to distinguish two main frequencies: the resonant frequency ($f_{r}$), where the admittance was maximised at 2.56 GHz, and the anti-resonant frequency ($f_{a}$), where the same was minimised at 2.62 GHz. Figure 1d shows the modal shape of a confined standing wave evaluated at the resonance. The wave travelled vertically in the piezoelectric and had its maximum displacement at the electrodes, as was expected.

## 3. Fabrication Protocol

**Figure 2:**
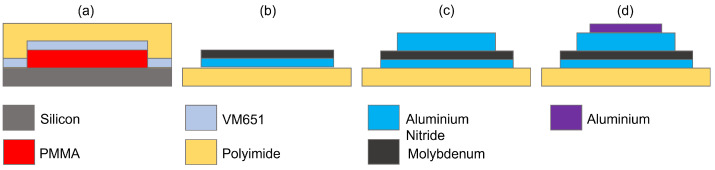
Fabrication steps for the resonator: (**a**) substrate preparation, (**b**) bottom electrode patterning, (**c**) piezoelectric deposition, (**d**) top electrode patterning.

The fabrication process of the flexible FBAR is detailed in Figure 2. As a first step, a sacrificial PMMA layer was spin-coated on a donor silicon wafer. The role of the sacrificial layer was to allow the detachment of the polyimide substrate at the end of the process. The edges of the wafer were cleaned of PMMA, and an adhesion promoter (VM651) was spin-coated and cured to reinforce the chemical bonding between the polyimide and silicon. This step enabled the formation of a solvent barrier and protected the PMMA in the centre of the wafer (see Figure 2a). The polyimide was spin-coated and cured. The recipes for the fabrication of the flexible substrate are fully detailed in Table 1.

Figure 2b sketches the formation of the bottom electrode, which was patterned by using a top-down approach. Specifically, two sputtering depositions of a 150 nm thick AlN interlayer and 200 nm of molybdenum were performed. The metal and the interlayer were patterned by using a positive resistance mask obtained through optical lithography and a BCl_3_-based inductively coupled plasma (ICP) etching. The piezoelectric layer was obtained by using the same approach, but in this case, a 1.2 μm AlN layer was deposited and patterned (see Figure 2c). The top electrode was fabricated by using a bottom-up approach consisting of inverse lithography, a sputtering deposition of 200 nm of aluminium, and lift-off of the metal (see Figure 2d). The recipes for the deposition and the pattering of the stack of the resonator are reported in Table 2. Finally, the barrier around the PMMA layer was removed by cutting the edges of the sacrificial layer with a blade; the sample was dipped in acetone until the complete detachment of the substrate occurred.

## 4. Results

**Figure 3:**
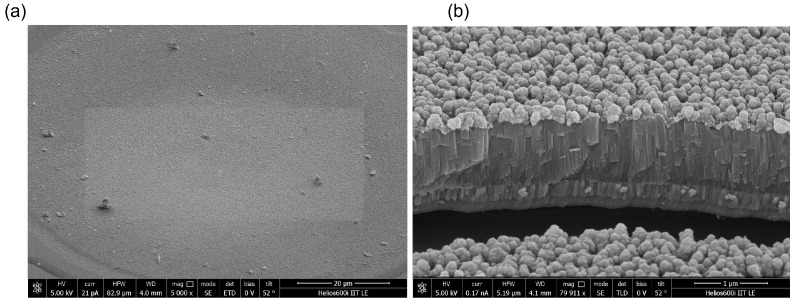
SEM acquisitions of the resonator: (**a**) top view of the device, (**b**) side view of the resonator.

Figure 3 illustrates the scanning electron microscope (SEM) acquisitions of the device. In particular, Figure 3a reports the top view of the resonator. The stack was patterned effectively, as the surface showed a clean profile without any inhomogeneities. The cross-section of the stack is shown in Figure 3b. As can be noted, the top electrode’s surface was clean and without any discontinuities. The structuring of the stack was effectively achieved, as no additional materials between the layers, e.g., organic compounds or resist residuals, were present.

**Figure 4:**
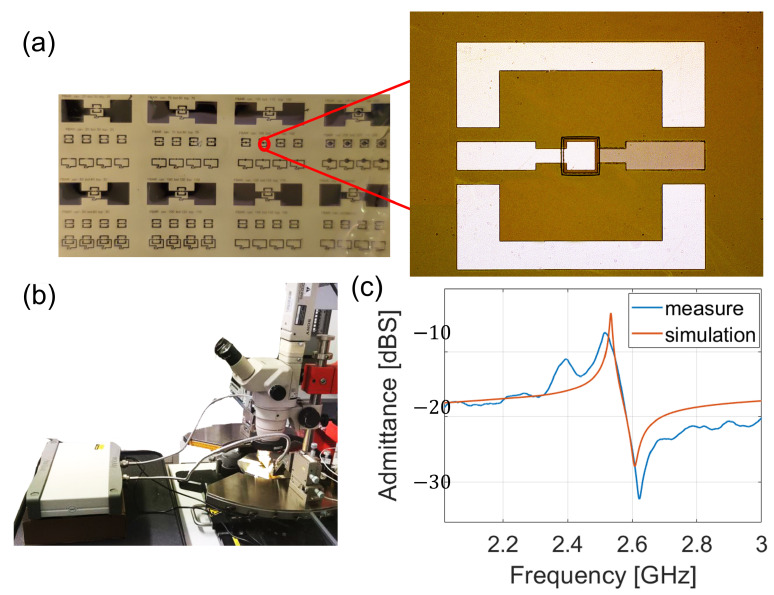
Characterisation of the FBAR. (**a**) Detached wafer and zoom on a resonator; (**b**) characterisation setup formed of VNA, probe station, and ground–signal–ground (GSG) probes; (**c**) comparison between simulations and measurements.

Figure 4 details the characterisation of the resonator. In particular, Figure 4a depicts the whole wafer with a zoom on a single resonator. The footprint of the FBAR was about 950 μm × 700 μm considering the resonating area and the feeding lines. The fabrication process proved to be very effective. The surface was flat despite the minimal residual tensile stress due to the deposition and patterning processes. The substrate withstood all of the fabrication steps, since no etched areas or delaminations of the metals were present. The resonator showed bright aluminium and molybdenum metals without any oxidised areas. The three lithographic processes were correctly performed, as no alignment errors were evident.

The FBAR was characterised in terms of admittance by using a vector network analyser and ground–signal–ground (GSG) with the setup illustrated in Figure 4b. The resonator was treated as a two-port device, where the input port was the top and the output port was the bottom electrode. The probes were aligned to the feeding lines by using a probe station with an optical microscope and connected to the VNA ports with SubMiniature version A (SMA) cables. The results are reported in Figure 4c. There was a maximum of admittance at 2.55 GHz, which corresponded to the resonance ($f_{r}$), while the anti-resonance dip ($f_{a}$) was placed at the frequency of 2.62 GHz. As can be noted, there was an optimal agreement with the FEM simulation with a minimum error of 10 MHz between the resonance positions. The maximum and the minimum values of the admittance were higher in the simulated case because of losses introduced by the contact resistance. Moreover, the measure presented small oscillations that could have been due to the bending of the cables when connecting the probes with the VNA. The resonators presented a maximum Q-factor (*Q*) and electromechanical coupling factor ($k_{eff}^{2}$) of 218 and 6.0%, respectively, which were evaluated as follows:

(2) $$Q=f/\Delta f_{|3dB}$$

(3) $$k_{eff}^{2}={\left(\frac{\pi }{2}\right)}^{2}\frac{\left(f_{a}-f_{r}\right)}{f_{r}}$$

Table 3 compares the advantages of our device with the state of the art of FBARs on flexible substrates.

As can be seen, our resonator presented the highest electromechanical coupling factor combined with the lowest number of fabrication steps. Unlike the device in [22,25], our resonator did not exploit a suspended structure. The confinement of the standing wave in the piezoelectric region was obtained by exploiting a molybdenum/polyimide acoustic interface. This procedure allowed cheaper and faster fabrication. Moreover, the absence of membranes allowed the formation of cracks to be avoided at the cost of a lower reflectivity and a lower Q-factor.

**Figure 5:**
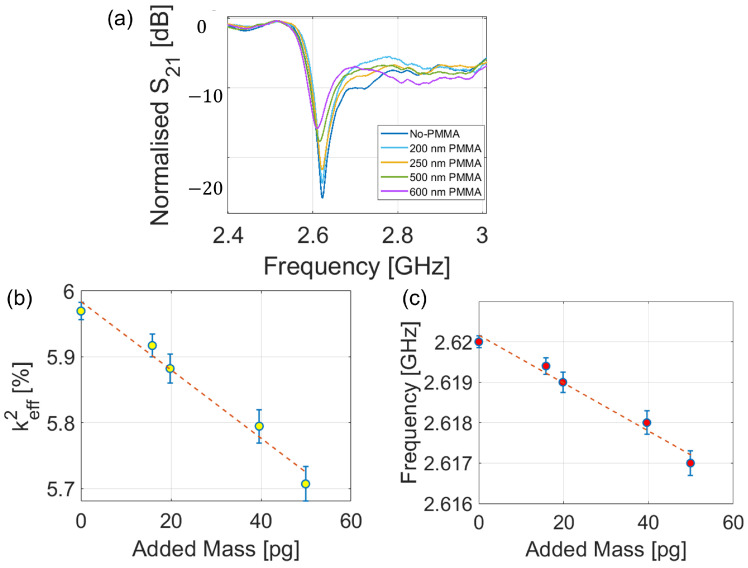
Experiment on the mass sensitivity of the FBAR. (**a**) Scattering parameter *S*_21_ for different PMMA layer thicknesses, (**b**) $k_{eff}^{2}$ versus added PMMA mass, and (**c**) frequency shift versus PMMA mass.

The mass sensitivity of the resonator and its feasibility as a gravimetric sensor were proved through the spin-coating deposition of PMMA layers with thicknesses from 0 to 650 nm on top of the FBAR. The response of the resonator was measured for all of the mass loadings, and the results are shown in Figure 5. As can be noted, the more mass was deposited on the top electrode, the lower the working frequency became because of the mass-loading effect. Moreover, as highlighted in Figure 5b, the addition of PMMA increased the dielectric losses of the resonator, thus flattening the dip of the scattering parameter $S_{21}$ and decreasing the electromechanical coupling factor. Finally, the calibration of the sensor (Figure 5c) was obtained from the polynomial fitting of the experimental results, whose expression is detailed in Equation (4).

(4) $$f_{a}\left(m\right)=2.62-23.31m\left[\frac{ppm}{pg}\right]$$

A linear dependence between mass loading and the anti-resonance value could be observed, proving the feasibility of the resonator as a gravimetric sensor with a responsivity of about 23 ppm/pg.

## 5. Conclusions

The fabrication of flexible MEMS resonators that work in the GHz range is a crucial challenge to overcome in the quest for wearable and wireless sensors. In this work, a gravimetric sensor based on a flexible AlN-based FBAR resonator was presented. The device was designed by using an FEM model to estimate the electrical and mechanical responses. The high effectiveness of the fabrication protocol was demonstrated, as it resulted in an optimal-quality resonator with a response that was perfectly in line with the simulation results. The resonator showed an optimal performance with a Q-factor of 218 and an electromechanical coupling factor of 6%. In addition, the mass responsivity of the resonator was estimated by spinning several amounts of PMMA in layers that were from 0 to 650 nm thick. The device was demonstrated to have a linear dependence on mass variation, thus proving its suitability as a gravimetric sensor. The sensitivity was evaluated, and a promising value of 23.31 ppm/pg was obtained. We strongly believe that these results can pave the way to a new class of wearable MEMS sensors that exploit UHF transmissions.

## Figures and Tables

**Table 1:** Process parameters of the polyimide and sacrificial layer.

	PMMA	Polyimide
Spin Velocity [RPM]	1500	1500
First Curing Temperature [°C]	180	130
First Curing Time	0 h:2^′^0^″^	1 h:0^′^0^″^
Second Curing Temperature [°C]	-	200
Second Curing Time	-	2h:00^′^0^″^

**Table 2:** Process parameters of the FBAR stack.

	Interlayer	Bottom	Piezo	Top
Sputtering Deposition				
Base Pressure [mBar]	$10^{-8}$	$10^{-8}$	$10^{-8}$	$10^{-8}$
Power [W]	1000	200	1250	400
Time	8.30^′^	27^′^	46^′^	7^′^20^″^
Optical Lithography				
Spin velocity [rpm]	-	2000	2000	2000
Temperature of the Pre-Exposure Bake [°C]	-	110	110	110
Time of the Pre-Exposure Bake	-	1^′^	1^′^	1^′^
First exposure [mJ/cm^2^]	-	140	140	100
Temperature of the Post-Exposure Bake [°C]	-	-	-	120
Flood Exposure [mJ/cm^2^]	-	-	-	700
Developing	-	1.30^′^	1.30^′^	30^″^
ICP etching				
Gas Concentrations [sccm]: BCl${}_{3}^{1}$, Ar^2^	100^1^, 25^2^	45^1^, 20^2^	100^1^, 25^2^	-
Time	5^′^	5^′^	25^′^	-

**Table 3:** Comparison of flexible FBARs.

	$k_{\mathrm{eff}}^{2}\left[\%\right]$	Fabrication Steps	Membrane	Substrate Material
[22]	3.1	8	Yes	thin-silicon
[26]	3.1	5	No	Polyimide
[25]	5.1	8	Yes	PET
**This work**	6.0	5	No	Polyimide

## Data Availability

Not applicable.
